# Warfarin Anticoagulation Exacerbates the Risk of Hemorrhagic Transformation after rt-PA Treatment in Experimental Stroke: Therapeutic Potential of PCC

**DOI:** 10.1371/journal.pone.0026087

**Published:** 2011-10-19

**Authors:** Waltraud Pfeilschifter, Daniel Spitzer, Josef Pfeilschifter, Helmuth Steinmetz, Christian Foerch

**Affiliations:** 1 Department of Neurology, Goethe University Hospital, Frankfurt am Main, Germany; 2 Department of General Pharmacology and Toxicology, Goethe University Hospital, Frankfurt am Main, Germany; University of South Florida, United States of America

## Abstract

**Background:**

Oral anticoagulant therapy (OAT) with warfarin is the standard of stroke prevention in patients with atrial fibrillation. Approximately 30% of patients with cardioembolic strokes are on OAT at the time of symptom onset. We investigated whether warfarin exacerbates the risk of thrombolysis-associated hemorrhagic transformation (HT) in a mouse model of ischemic stroke.

**Methods:**

62 C57BL/6 mice were used for this study. To achieve effective anticoagulation, warfarin was administered orally. We performed right middle cerebral artery occlusion (MCAO) for 3 h and assessed functional deficit and HT blood volume after 24 h.

**Results:**

In non-anticoagulated mice, treatment with rt-PA (10 mg/kg i.v.) after 3 h MCAO led to a 5-fold higher degree of HT compared to vehicle-treated controls (4.0±0.5 µl vs. 0.8±0.1, p<0.001). Mice on warfarin revealed larger amounts of HT after rt-PA treatment in comparison to non-anticoagulated mice (9.2±3.2 µl vs. 2.8±1.0, p<0.05). The rapid reversal of anticoagulation by means of prothrombin complex concentrates (PCC, 100 IU/kg) at the end of the 3 h MCAO period, but prior to rt-PA administration, neutralized the exacerbated risk of HT as compared to sham-treated controls (3.8±0.7 µl vs. 15.0±3.8, p<0.001).

**Conclusion:**

In view of the vastly increased risk of HT, it seems to be justified to withhold tPA therapy in effectively anticoagulated patients with acute ischemic stroke. The rapid reversal of anticoagulation with PCC prior to tPA application reduces the risk attributed to warfarin pretreatment and may constitute an interesting therapeutic option.

## Introduction

Intravenous thrombolysis with recombinant tissue plasminogen activator (rt-PA) reduces death and disability when administered within the first 4.5 h after symptom onset in patients with ischemic stroke, although the pooled data of the large thrombolysis in stroke trials (ECASS, ATLANTIS, NINDS, and EPITHET) revealed that the risk of symptomatic intracerebral hemorrhage is considerably elevated in rt-PA patients (5.2%) as compared to controls (1.0%) [1]. In general, hemorrhagic transformation (HT) of ischemic brain tissue after rt-PA therapy is frequently observed and can be graded into hemorrhagic infarction (petechial infarct without space-occupying effect) or parenchymal hematoma (hemorrhage with mass effect) [2].

It is believed that the risk of HT is increased in patients with a compromised blood coagulation system. Therefore, the AHA guidelines for the management of stroke recommend withholding thrombolysis with from patients being under oral anticoagulant therapy (OAT) with an international normalized ratio (INR)>1.7 [3]. However, there are only few clinical and no experimental data to substantiate the assumption that effective warfarin treatment exacerbates the risk of thrombolysis-associated intracerebral hemorrhage. While it is widely accepted that patients with a compromised blood coagulation system due to effective anticoagulation or a hemostatic disorder should not be treated with systemic thrombolysis, the issue is less clear for patients with subtherapeutic INR values. A recent, vividly debated report on 107 warfarin-treated stroke patients who were eligible for rt-PA treatment due to subtherapeutic INR values of <1.7 showed an increased rate of symptomatic intracerebral hemorrhage in the warfarin-treated group after thrombolysis even after multivariate adjustment for the relevant covariates age, atrial fibrillation, initial stroke severity by the National Institutes of Health Stroke Scale, and INR [4]. A large observational study assessing the influence of antithrombotic pretreatment on stroke severity in patients with ischemic stroke and atrial fibrillation found no difference in the frequency of spontaneous secondary intracranial hemorrhage in the warfarin-treated group compared to the group not receiving anticoagulant treatment prior to admission [5]. However, the ratio of hemorrhagic transformation secondary to thrombolysis in the anticoagulated group was much higher compared with the antiplatelet-treated and antithrombotic-naïve patients [6].

Based on a mouse model of focal cerebral ischemia, we have recently shown that large hemispheric infarctions evolving under effective warfarin treatment reveal significantly enlarged amounts of HT as compared to those evolving under normal coagulation. This applies to both the upper and the lower end of the therapeutic INR range [7]. The present study investigates whether thrombolytic treatment with i.v. rt-PA further exacerbates the risk of HT in warfarin-anticoagulated mice, with the aim to gain *in vivo* evidence for the widespread practice to withhold thrombolysis in anticoagulated stroke patients. Furthermore, we evaluated whether the rapid reversal of anticoagulation using prothrombin complex concentrates (PCC) just before rt-PA administration reduces the risk of excess HT caused by effective warfarin treatment.

## Results

### Both treatment with rt-PA and effective oral anticoagulation harbor a significant risk of hemorrhagic transformation

**Figure 1:**
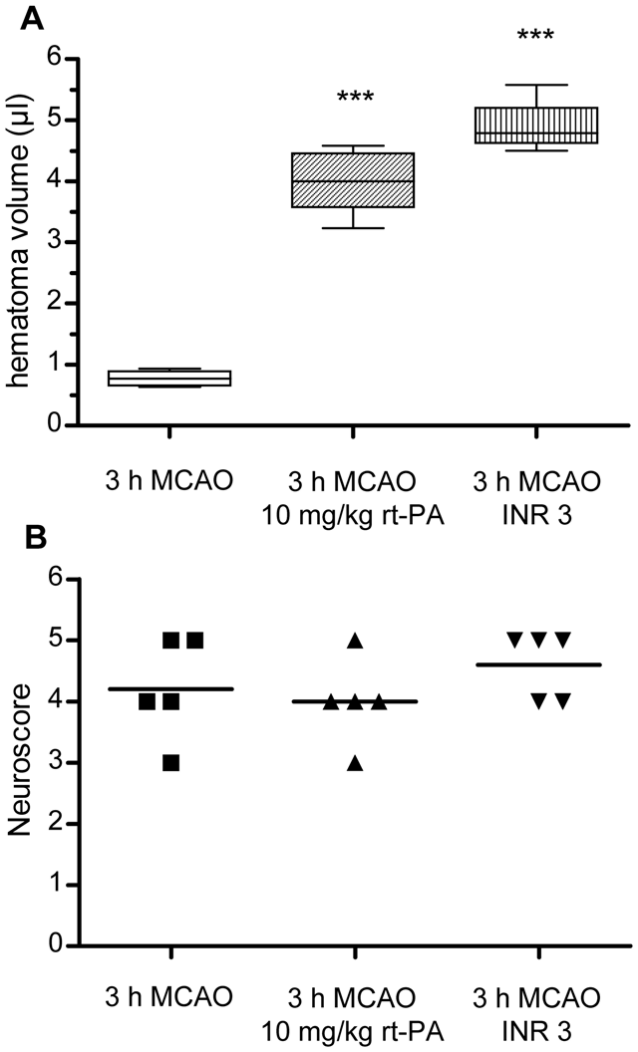
Comparison of hemorrhagic transformation 24 h after MCAO in mice with different antithrombotic pretreatment. A) Comparison of HT volumes in mice who received i.v. thrombolysis with 10 mg/kg human recombinant t-PA directly prior to reperfusion (middle, n = 5) and mice who were effectively anticoagulated to an INR of approximately 3 at the onset of 3 h MCAO (right, n = 4 after exclusion of outlier, see Fig. S1) with mice who underwent 3 h MCAO without any treatment (left, n = 5). Hematoma volume was determined by a hemoglobin assay. Data are depicted as box and whiskers plots showing the 25 to 75 interquartile range and the extreme values. Statistical significance was analyzed with ANOVA and bonferroni correction for multiple testing. *** p<0.001. B) Neuroscores were assessed for statistical significance with a Kruskal-Wallis test and Dunn's correction. Differences between groups were not significant (median values 4, 4 and 5).

After 3 h MCAO, leading to ischemic lesions of 111.6±35.4 mm^3^ in our hands (n = 6, data not shown), the extent of HT in untreated animals was found to be very low (0.8±0.1 µl, n = 5) (Fig. 1A). In mice without prior warfarin treatment mice receiving i.v. rt-PA (10 mg/ml) immediately prior to reperfusion, the degree of HT increased five-fold (4.0±0.5 µl, n = 5, p<0.001 compared to vehicle-treated mice) (Fig. 1A). Mice pretreated with warfarin to an INR of approximately 3 prior to MCAO also showed a significantly higher degree of HT compared to control mice (4.9±0.5 µl, n = 4, p<0.001 compared to anticoagulation-naïve mice) (Fig. 1A). After 3 h MCAO, all mice showed a severe neurological deficit, and no differences between groups were observed (Fig. 1B). One mouse in the control group and and one mouse in the warfarin group had to be excluded due to failure of i.v. substance application (vehicle in both cases). Corresponding mice were substituted to the operator in a blinded fashion. Post-hoc statistical power analyses showed an effect size (Cohen's d) of 5.77 for the comparison of control mice with mice receiving rt-PA and of 7.25 for the comparison of control mice with warfarin-treated mice both providing a statistical power of 100%.

### Effective warfarin anticoagulation exacerbates the risk of hemorrhagic transformation after rt-PA treatment

**Figure 2:**
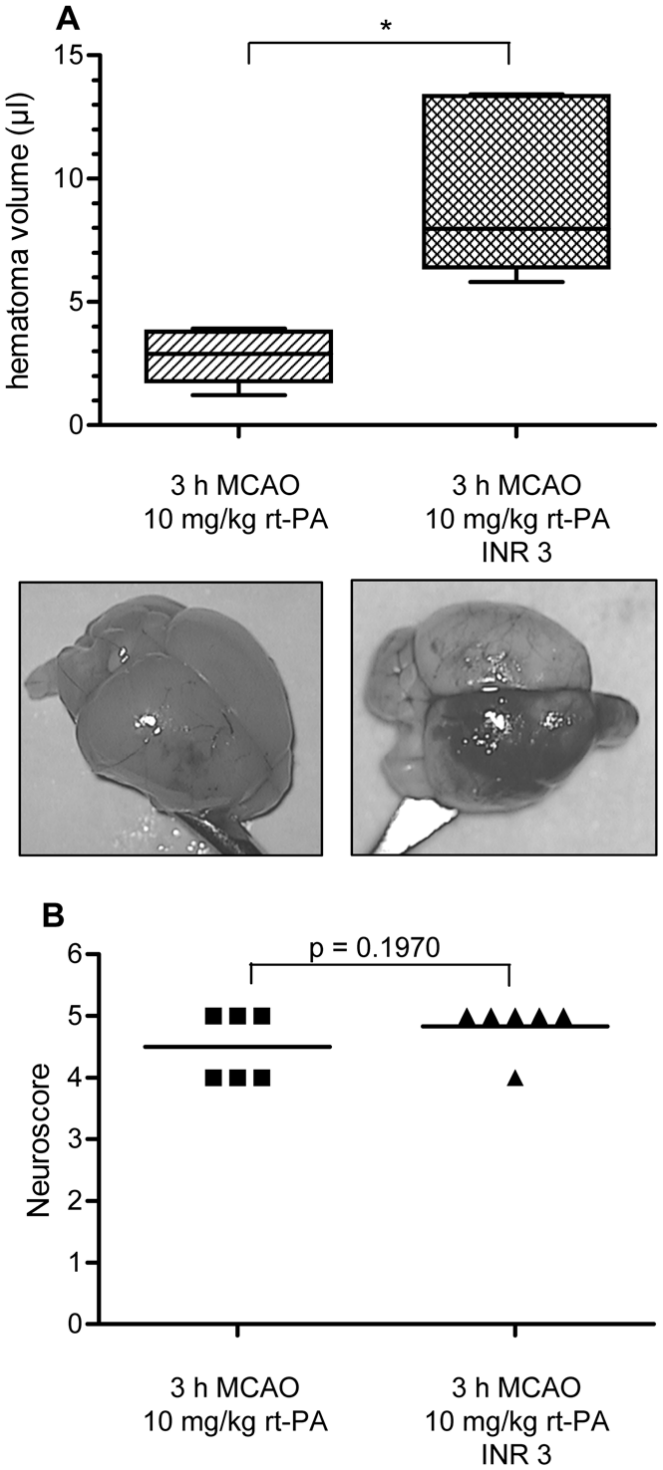
Hemorrhagic transformation after i.v. thrombolysis with rt-PA is excessively increased in effectively anticoagulated mice. A) Mice without prior warfarin intake subjected to 3 h MCAO were treated with 10 mg/kg human rt-PA directly prior to reperfusion (left, n = 5). In comparison, mice anticoagulated to an INR of approximately 3 at the onset of cerebral ischemia (right, n = 5) showed a vast increase in hematoma volume after t-PA treatment. Statistical significance was essayed with student's unpaired, two-tailed t-test. ** p<0.01. B) Neuroscores were analyzed for statistical significance with a one-tailed Mann Whitney test and the p-value given as a Gaussian approximation (median values 4.5 and 5, p = 0.1970).

When comparing the effects of i.v. rt-PA treatment after 3 h MCAO in non-anticoagulated mice and in mice pretreated with oral warfarin to an INR of approximately 3, warfarin pretreatment vastly increased the amount of HT (9.2±3.2 µl vs. 2.8±1 µl, n = 6 in both groups, p<0.05) (Fig. 2A). There was a tendency, however non-significant, towards a more severe neurological deficit in warfarin-treated mice that received rt-PA as compared to mice without prior warfarin intake that received rt-PA (median 5 vs. 4.5, p = 0.1970 [Gaussian approximation]) (Fig. 2B). One mouse receiving rt-PA without prior warfarin treatment had to be excluded due to a failure of i.v. substance application and was substituted to the operator in a blinded fashion. Post-hoc statistical power analyses showed an effect size (Cohen's d) of 4.36 for this comparison providing a statistical power of 99%.

### Rapid reversal of warfarin anticoagulation with PCC reduces the excessive risk of hemorrhagic transformation associated with rt-PA treatment after MCAO in anticoagulated mice

**Figure 3:**
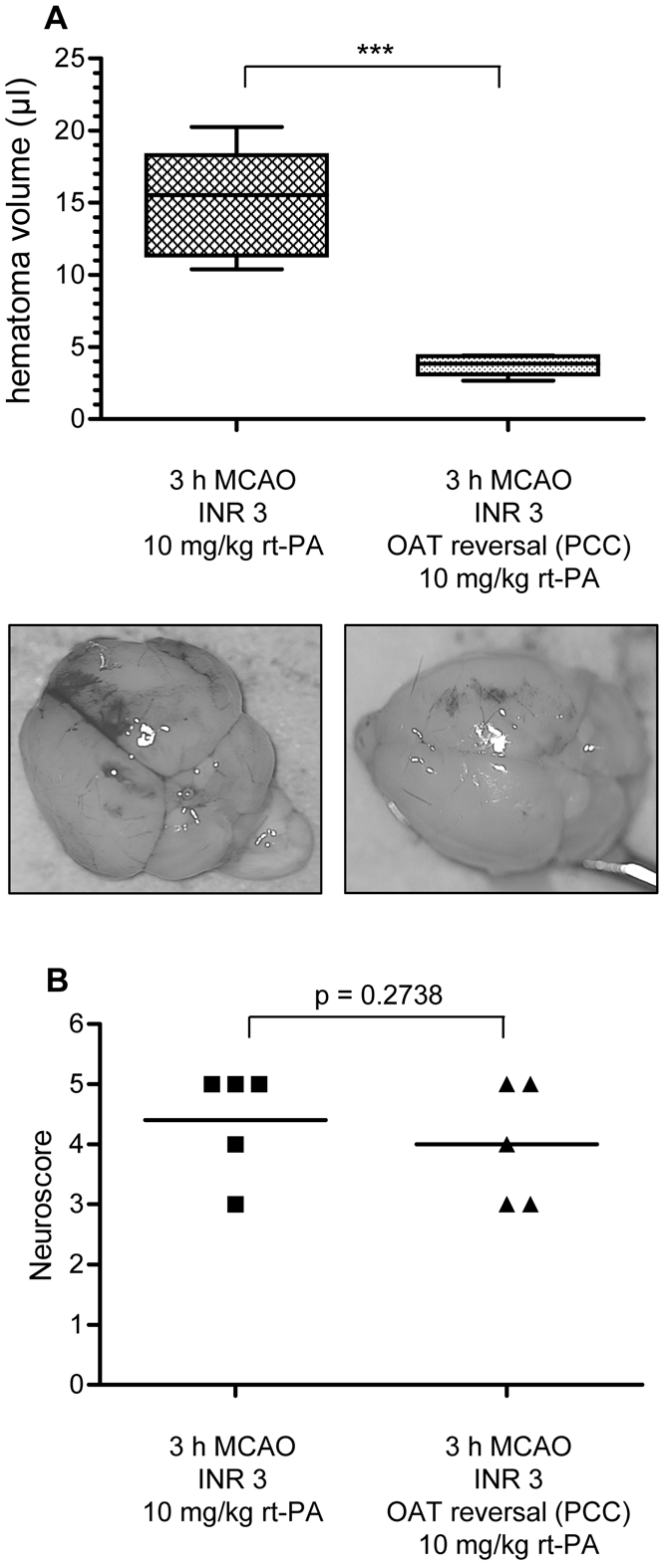
Reversal of anticoagulation with PCC diminishes the excessive rt-PA-associated risk of hemorrhagic transformation in warfarin-treated mice. A) Anticoagulated mice treated with i.v. rt-PA (10 mg/kg) after 3 h MCAO develop large intracerebral hematomas (left, n = 5). Reversal of oral anticoagulation with i.v. PCC (100 IU/kg) directly before the administration of rt-PA counterbalances the excessive risk of hemorrhagic transformation (right, n = 5). Statistical significance was essayed with student's unpaired, two-tailed t-test. ** p<0.001. B) Neuroscores were analyzed for statistical significance with a one-tailed Mann Whitney test and the p-value given as a Gaussian approximation (median values 5 and 4, p = 0.2738).

In effectively anticoagulated mice receiving i.v. rt-PA treatment after 3 h MCAO, the rapid reversal of anticoagulation by means of PCC application directly prior to rt-PA administration significantly reduced the amount of HT as compared to non PCC-treated mice (3.8±0.7 µl, n = 5 in all groups vs. 15.0±3.8 µl, p<0.001) (Fig. 3A). Restoring the coagulation system with PCC reduced the extent of HT to values comparable with those seen in rt-PA treated animals without prior warfarin treatment (2.8±1.0 µl vs. 3.8±0.7 µl, p = 0.12, left column in Fig. 2 and right column in Fig. 3A). In terms of neurological function, mice whose anticoagulation was reversed with PCC showed a non-significant tendency towards a better functional outcome (median 5 vs. 4, p = 0.2738 [Gaussian approximation]) (Fig. 3B). Two mice in the group receiving rt-PA after prior warfarin treatment without anticoagulation reversal with PCC died due to major intracranial hemorrhage including vast subarachnoid hemorrhage. Two mice in the PCC-group had to be excluded due to a failure of i.v. substance application and were substituted to the operator in a blinded fashion. Post-hoc statistical power analyses showed an effect size (Cohen's d) of 7.47 for this comparison providing a statistical power of 100%.

### Prevention of excessive HT after rt-PA treatment in mice with prior warfarin intake leads to a less severe neurological deficit

**Figure 4:**
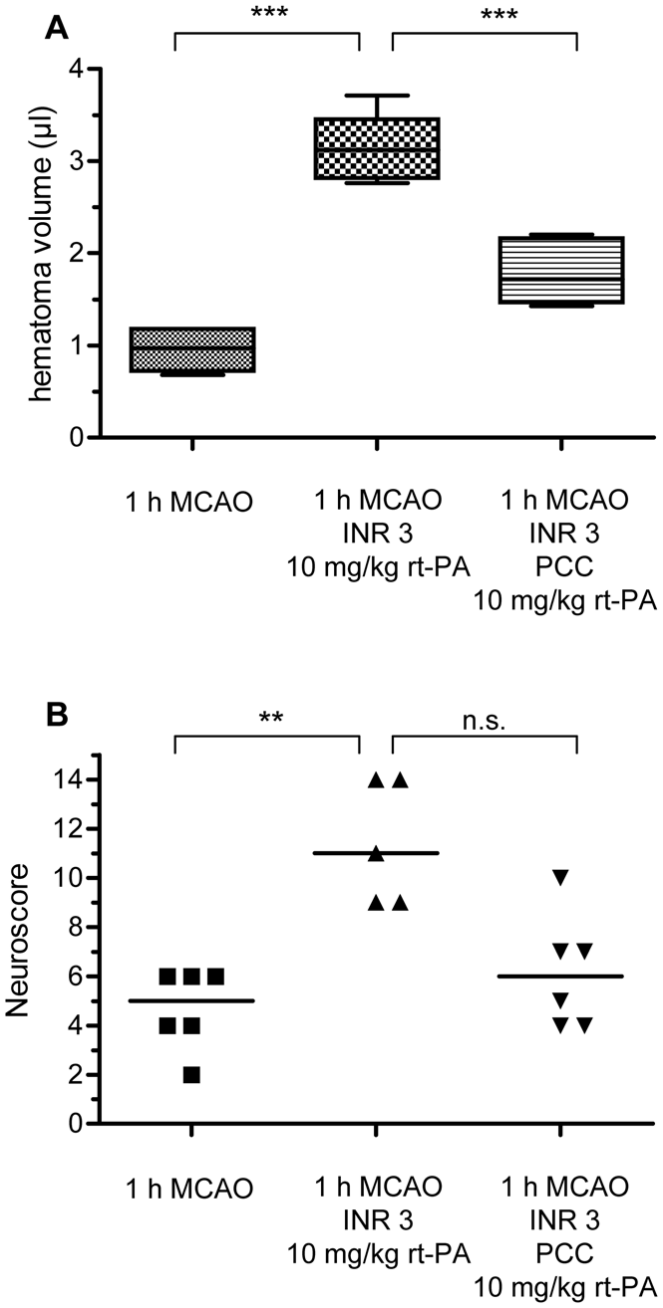
The excessive degree of HT and the reversal of anticoagulation with PCC are functionally relevant in a model of milder strokes. A) Mice receiving rt-PA (10 mg/kg) after 1 h MCAO under warfarin treatment (middle, n = 5) show a significantly greater HT volume than control mice (left, n = 6). Reversal of oral anticoagulation with PCC (100 IU/kg) significantly attenuates HT (right, n = 6). *** p<0.001. B) Functional neurological outcome was evaluated on a 14-point neuroscore and statistical significance was assessed with a Kruskal-Wallis test with Dunn's correction, median values are given (5, 11 and 6, ** p<0.01).

After 1 h MCAO, leading to ischemic lesions of 49.7±25.1 mm^3^ in our hands (n = 6, data not shown), mice with prior warfarin treatment who received rt-PA at the onset of reperfusion also developed a considerable degree of HT (3.1 µl±0.4, n = 5 vs. 1.0±0.2, in control mice, n = 6, p<0.001) (Fig. 4A). Reversal of warfarin anticoagulation with PCC prior to rt-PA treatment reduced the HT volume considerably (1.7±0.3 µl, n = 6, p<0.001 compared to anticoagulated and rt-PA-treated without restoration of the coagulation system) (Fig. 4A). In this model of milder stroke, the increase of HT in rt-PA-treated mice with prior warfarin anticoagulation translated into a relevant deterioration of the functional neurological outcome (median neuroscore of 11 vs. 5 in control mice, p = 0.01 [Gaussian approximation]). Functional outcome showed a clear, however statistically non-significant tendency to be improved in the mice who were substituted with PCC before they received rt-PA (median neuroscore 6, p>0.05 [Gaussian approximation]) (Fig. 4B). One mouse was excluded from the group with warfarin pretreatment prior to rt-PA due to death secondary to an abdominal bleeding. This mouse was not substituted to the operator. Post-hoc statistical power analyses showed an effect size (Cohen's d) of 5.28 for the comparison of control mice with mice receiving rt-PA after warfarin pretreatment providing a statistical power of 99% and of 2.33 for the comparison of mice receiving rt-PA after warfarin pretreatment with equally-treated mice who had their anticoagulation reversed with PCC prior to rt-PA providing a statistical power of 90%.

## Discussion

In this experimental study, we model for the first time the consequences of administering rt-PA in ischemic stroke occurring under effective warfarin anticoagulation. The extent of HT after focal cerebral ischemia is increased in mice that are under warfarin treatment at the onset of ischemia and further potentiated by treatment with rt-PA.

Similar to the findings of our previous experimental study [7], we observed that mice without prior warfarin treatment develop very little HT after 3 h MCAO, despite the evolution of large territorial infarctions. In contrast, MCAO performed in effectively anticoagulated mice leads to a considerable amount of HT both at the low and the high end of the therapeutic INR range of 2–3 [7]. Since we have previously shown that the OAT-associated increase in HT after cerebral ischemia largely depends on reperfusion [7], we assume that the compromised coagulation system leads to a prolongation and hence increase of microbleeds which occur naturally at the blood-brain barrier in the course of a large hemispheric infarction.

Furthermore, we showed that administering rt-PA just before reperfusion in mice without prior warfarin treatment subjected to 3 h of MCAO also leads to a significant increase of HT as compared to untreated controls. These findings reflect the results of clinical trials of rt-PA in stroke that show a rise in the incidence of symptomatic secondary intracerebral hemorrhage about tenfold compared to untreated patients, although our study does not capture hemorrhage incidence but hemorrhage volume [1]. The increase in HT after rt-PA treatment is a well-known feature of this drug which is due to pleiotropic properties which include the proteolytic activation of other proteases like matrix metalloproteinases (MMP, especially MMP-9) and NMDA receptor-associated excitotoxicity [8].

Compared to non-anticoagulated animals that received rt-PA after 3 h MCAO, mice that were effectively anticoagulated with warfarin during this experimental paradigm revealed a significantly higher degree of HT. Thus, whereas both effective anticoagulation and rt-PA treatment *per se* increase the amount of HT in ischemic stroke, the combination of both further exacerbates the extent of HT. Mechanistically, the serine-protease rt-PA catalyzes the generation of plasmin from plasminogen in a thrombus-specific manner. This is achieved by the fibrin-enhanceability of its enzymatic activity which leads to only a limited conversion of plasminogen in the absence of a fibrin-rich clot [9]. Oral anticoagulation with the vitamin K antagonist warfarin, leading to a depletion of the vitamin K-dependent coagulation factors II, VII, IX, and X, does not directly interfere with the activity of rt-PA or plasmin nor does it have a direct effect on an established thrombus [10]. Cerebral ischemia leads to a breakdown of the blood-brain barrier that is fuelled by reperfusion injury and administration of rt-PA [11]. We have shown previously that reperfusion strongly determines the amount of HT in mice who experience cerebral ischemia under effective OAT [7]. Therefore we assume that rather than interfering directly with the activity of rt-PA or plasminogen, the impaired plasmatic hemostasis in case of warfarin anticoagulation leads to a propagation of initially minor bleedings occurring at the blood-brain barrier that is itself compromised by both ischemia/reperfusion injury and tPA-mediated injury. Since all mice in our model experienced some degree of HT, we cannot delineate whether warfarin pretreatment increases the risk of HT on its own (i.e. the *de novo* occurrence of HT) or just increases the volume of bleeding.

Our results strongly support the guidelines of acute stroke treatment who advise withholding thrombolytic treatment from patients under effective oral anticoagulation. *Clinical* data from the aforementioned study [4] of warfarin-treated patients with a subtherapeutic INR<1.7 who were treated with i.v. thrombolysis according to the AHA guidelines point into the same direction. In contrast, another very recent South Korean analysis did not find a significant influence of OAT with a subtherapeutic INR<1.7 on the rate of symptomatic intracerebral hemorrhages after rt-PA treatment [12]. One limitation of both studies was the small number of 13 and 28 patients, respectively [4], [12].

While investigating the potential of rapidly reversing anticoagulation by PCC as a therapeutic intervention to limit the exceeding risk of HT in anticoagulated mice receiving rt-PA in focal cerebral ischemia, we found a strong reduction of HT to levels comparable with those of OAT-naïve mice treated with rt-PA. This finding further emphasizes the role of an intact plasmatic coagulation cascade in preventing rt-PA associated HT. Whether OAT can be safely reversed in patients with acute ischemic stroke without provoking additional thrombus formation and vessel occlusion has yet to be investigated. One small single-center observational study suggests that the treatment of patients with INR values at the lower end of the therapeutic range with fresh frozen plasma and the subsequent catheter-based intra-arterial thrombolysis with reteplase is possible without development of intracranial hemorrhage in these cases. Two of the patients experienced early neurological improvement [13]. Addressing the widespread concern of an over-activation of the coagulation system after anticoagulation reversal, two small observational studies with a total of 85 non-stroke patients requiring immediate reversal of OAT treatment have shown no activation of the coagulation system as documented by plasma fibrinogen, D-dimer and platelet count. However,two cases of severe thrombotic events occurred, in one patient predisposed by a metastatic cancer and in the other patient associated with sepsis and a beginning disseminated intravascular coagulation [14], [15]. It would be of utmost importance to know whether it is safe to restore the impaired plasmatic coagulation cascade in patients with ischemic stroke occurring under OAT. This may help this group of patients to benefit from systemic or local thrombolysis.

After 3 h MCAO, all mice typically show large territorial infarctions leading to severe functional impairment with a median neuroscore of 4. There was a tendency, however non-significant, towards a more severe neurological deficit in OAT mice that received rt-PA as compared to non-anticoagulated mice that received rt-PA. Anticoagulated mice receiving PCC before thrombolysis showed a tendency towards a better outcome than OAT mice who received rt-PA without reversal of OAT but regrettably, our study, whose primary endpoint was HT volume, was underpowered to detect significant differences in functional outcome in this model. To evaluate the modulation of the functional outcome by conditions which either increase HT (i.e., warfarin anticoagulation) or decrease HT (i.e., anticoagulation reversal prior to rt-PA therapy), we verified the most important findings of our study in a model of milder stroke with a reduced MCA occlusion time of 1 h. In this experiment, we found that the increase in HT which was seen in the group that received rt-PA after 1 h MCAO under warfarin-pretreatment translated into a less favourable outcome while the substitution of PCC in this setting did not worsen outcome but led to a strong tendency towards a better outcome corresponding with the reduction in HT. Since we did not assess ischemic lesion size, we cannot exclude that the administration of PCC lead to an increase in ischemic lesion size. However, our data on the neurological outcome argue against functionally relevant infarct growth after PCC treatment.

In our experiments, we used a therapeutically relevant time window, applying rt-PA directly prior to reperfusion following 3 h MCAO. Since rt-PA has a half-life in the circulation of approximately five minutes [16], we assume that it is enzymatically active when it reaches the ischemic brain area. We administered rt-PA as a single shot because the compromised coagulation in the warfarin-treated mice did not allow a permanent venous access which would have been necessary for a continuous infusion over 1 h.

In summary, our experimental study in mice shows that rt-PA associated HT after focal cerebral ischemia is potentiated by prior OAT, and therefore provides in-vivo evidence for witholding rt-PA treatment in effectively anticoagulated patients. Reversal of OAT with PCC might be a therapeutic option to render recanalization therapy possible in selected patients.

## Methods

### Mouse model of oral anticoagulation

Male C57BL/6 mice (10 weeks old, strain J) were used in accordance with the National Institute of Health Guide for the Care and Use of Laboratory Animals (NIH Publications No. 80-23, revised 1996). The experiments were approved by the local governmental authorities (Regierungspraesidium Darmstadt, Germany, approval number F143/34). Following a recently established protocol, we administered warfarin via oral uptake through bottled drinking water. In brief, a 5 mg coumadin tablet (warfarin sodium, crystalline, Bristol Myers Squibb, Munich, Germany) was dissolved in 375 ml tap water. Assuming a body weight of 20 g and a water consumption of 15 ml/100 g per 24 h, this dosage corresponds to a warfarin uptake of 0.040 mg (2.00 mg/kg) per mouse for a 24 h feeding period, a dosage which has been shown to result in a mean INR value of 2.9±0.9 in our previous study [7]. In the same study, we measured a mean INR of 0.9±0.1 in non-anticoagulated mice. Details of this mouse model of oral anticoagulation can be found elsewhere [17]. After warfarin withdrawal, INR values remain stable for the next 6 h and drop to normal values within 24 h. All four vitamin K dependent coagulation factors are diminished in this mouse model, thereby mimicking full warfarin anticoagulation [18].

### Middle cerebral artery occlusion

The operator was blinded to the treatment status of the animals. Transient middle cerebral artery occlusion (MCAO) was performed as described previously [19]. Mice were anaesthetized with 1.5% isoflurane (Forene; Abbott, Wiesbaden, Germany) and 0.1 mg/kg buprenorphine (Temgesic; Essex Pharma, Munich, Germany) under spontaneous respiration. Focal cerebral ischemia was induced by introducing a silicone-coated 7-0 monofilament until it occluded the ostium of the right MCA. Regional cerebral blood flow was monitored by laser Doppler flowmetry (PF5010, Perimed, Sweden) to confirm vessel occlusion. The filament was withdrawn after 3 h or 1 h to allow reperfusion of the ischemic hemisphere. Following the operation, all animals received regular drinking water, withdrawing the warfarin supply of the previously anticoagulated animals. Animals were sacrificed at 24 h after assessment of their global neurological functions. Mice subjected to the longer occlusion period of 3 h who showed a severe neurological deficit were assessed with a 6 point neuroscore (0 = no deficit, 1 = failure to extend left forepaw, 2 = circling to the left, 3 = falling to the left, 4 = “barrel rolling”, 5 = unable to move spontaneously, 6 = dead). Mice subjected to the shorter occlusion period of 1 h were assessed by a more differentiated 14 point neuroscore modified from Chen et al [20]) (Table S1).

### Dosing and administration of rt-PA and PCC

Human recombinant tissue plasminogen activator (rt-PA; Actilyse®, Boehringer Ingelheim, Germany), which has been shown to be thrombolytically active in mice at the dose of 10 mg/kg was dissolved in aqua ad injectabilia at a concentration of 1 mg/ml and injected via the tail vein directly before reperfusion after 3 h MCAO in reanaesthetized mice. Human PCC (Prothromplex® 600 IE, Baxter, Vienna, Austria) was dissolved in aqua ad injectabilia and injected via the tail vein at a dosage of 100 IU/kg directly prior to the administration of rt-PA. We have shown previously that human PCC rapidly reverses warfarin anticoagulation in mice [18].

### Hemoglobin assay

For a quantitative analysis of HT, brains were homogenized 24 h after the induction of cerebral ischemia after transcardial perfusion with PBS. HT blood volume was measured using a photometric hemoglobin assay as previously described [17].

### Sample size calculation and Study design

Sample size calculation was based upon our previous study examining HT in vehicle or warfarin-treated mice subjected to the same stroke model [7]. In this study, untreated mice had shown a hematoma volume of 0.3±0.4 µl, whereas mice anticoagulated to an INR of approximately 3 had shown a hematoma volume of 5.2±2.7 µl. To detect meaningful changes in a similar experimental paradigm, a minimal sample size of four animals per group is required to detect this difference with a power (1 – β) of 0.9 and a level of acceptability of a false positive result (α) of 0.05 [21].

The study was performed in four steps. In a first step, we evaluated the degree of HT after a 3 h MCAO period in mice that either were on OAT or received i.v. rt-PA therapy immediately prior to reperfusion. Furthermore, we assessed the degree of HT after 3 h MCAO in vehicle-treated animals. 15 mice were randomized by the allocator in a 2∶1 ratio into a non-anticoagulated group and a group anticoagulated to an INR of approximately 3. After the MCAO procedure, the non-anticoagulated mice (whose status was known to the allocator) were injected with either rt-PA or vehicle by the operator in a blinded fashion.

In a second step, we assessed the effect of OAT on the extent of HT after i.v. rt-PA application immediately prior to reperfusion after 3 h MCAO. 12 mice were randomized in a 1∶1 ratio into a non-anticoagulated group and a group anticoagulated to an INR of approximately 3 and were operated in a blinded fashion.

In a third step, we evaluated whether the rapid reversal of anticoagulation in OAT mice reduces the extent of HT after treatment with i.v. rt-PA immediately prior to reperfusion after 3 h MCAO. 10 mice were anticoagulated to an INR of approximately 3. They were randomized in a 1∶1 ratio to receive either PCC or vehicle at the end of the 3 h MCAO period, Immediately thereafter, all mice were treated with i.v. rt-PA.

In a fourth step, we induced smaller ischemic lesions by a shorter MCAO occlusion time of 1 h to assess the functional impact of increased hemorrhagic transformation in less severly impaired mice. 10 mice were anticoagulated with warfarin and randomized in a 1∶1 ratio to receive either PCC or saline 30 minutes after the onset of cerebral ischemia. Directly prior to reperfusion, all ten mice received i.v. rt-PA. 5 mice without warfarin or rt-PA treatment served as controls.

Our predefined exclusion criteria were 1) lack of neurological deficit after 24 h (0 mice met this criterium), 2) death (met by 2 mice in the group of mice who received rt-PA after 3 h MCAO under warfarin, both mice showed large intraparenchymal and subarachnoid hemorrhages and 1 mouse in who received rt-PA after 1 h MCAO under warfarin) and 3) failure of intravenous application (met by 5 mice). In case of exclusion, the status of the animal was disclosed by the allocator and an equally treated mouse was substituted in a blinded fashion to the operator.

### Statistical analyses

Graph Pad Prism 4 (Graph Pad Software Inc., La Jolla, CA, USA) was used for statistical analysis. Results are given as mean ± SD and graphically presented as a box and whiskers plot depicting the mean, extreme values and the 25–75 interquartile range. Statistical significance was assessed with a one-way ANOVA with Bonferroni correction (multigroup analyses) and a two-tailed student's t-test (two-group analyses) for hematoma volume data. For non-parametric neuroscore data, we used a Kruskal-Wallis-test with Dunn's correction (multigroup analyses) and a Mann Whitney test with Gaussian approximation (two-group analyses). In the first part of our study, we observed one value which we suspected to be a significant outlier (Fig. S1). This was confirmed by the Grubb's test [22] and the outlier was excluded from the analysis.

## Supporting Information

Figure S1 **Exclusion of an outlier.** Hemorrhagic transformation after 3 h MCAO in mice who received i.v. thrombolysis with 10 mg/kg human recombinant t-PA directly prior to reperfusion (middle, n = 5) and mice who were effectively anticoagulated to an INR of approximately 3 at the onset of cerebral ischemia (right, n = 5) compared to control mice with MCAO without pretreatment (left, n = 5). Hematoma volume detected by the hemoglobin assay is given in µl for each animal. The extreme value of 14.7 µl in the MCAO at INR 3 group was identified as a significant outlier by the Grubb's test and excluded from the statistical analysis.
(TIF)

Table S1 **14 point neuroscore modified from Chen et al** [**20**] **.**
(DOCX)
