# Supplementary figures and images for: Warfarin Anticoagulation Exacerbates the Risk of Hemorrhagic Transformation after rt-PA Treatment in Experimental Stroke: Therapeutic Potential of PCC
Source: PLoS One. 2011 Oct 19;6(10):e26087. doi: 10.1371/journal.pone.0026087 (PMC3198453; doi:10.1371/journal.pone.0026087)

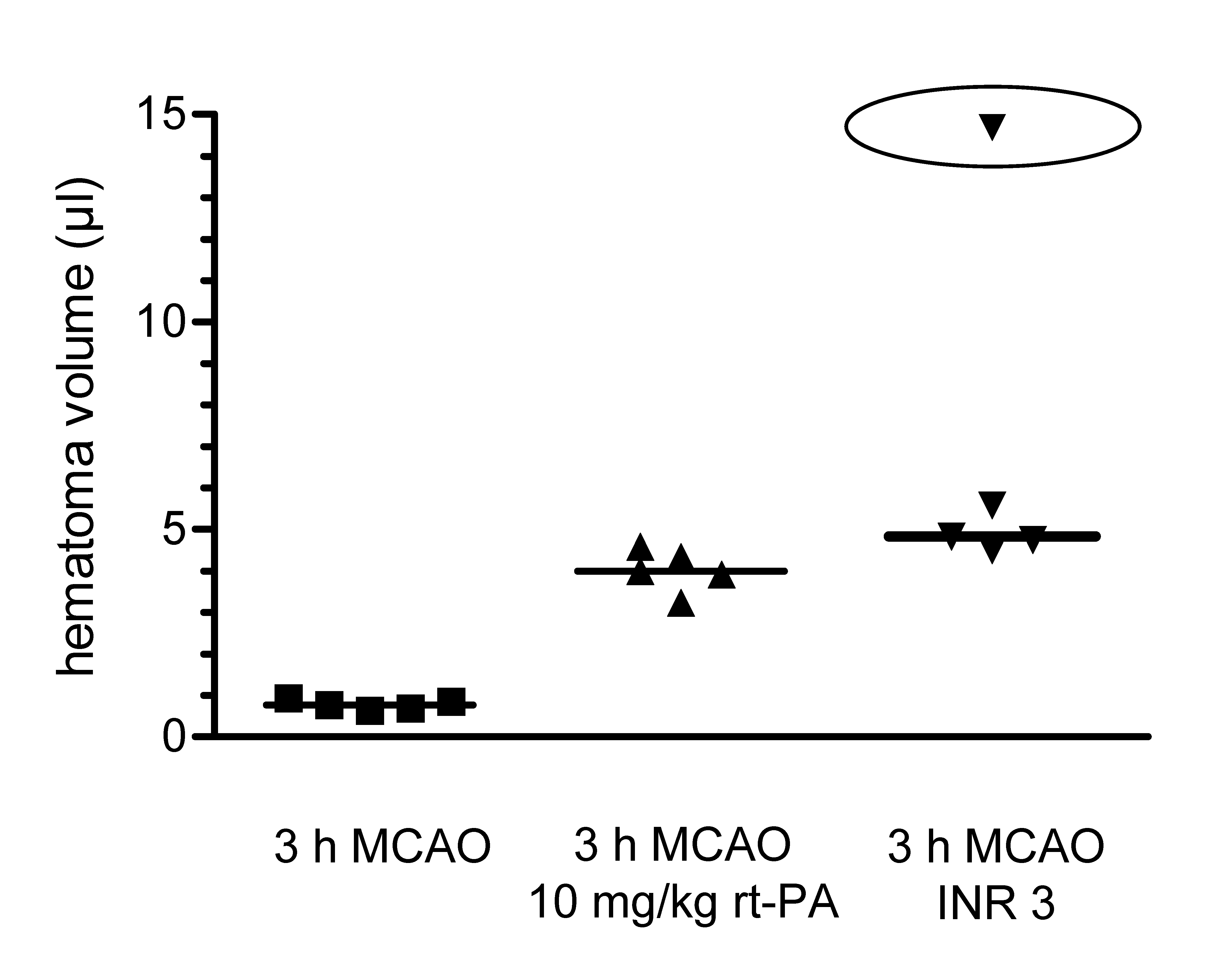

Supplement: Figure S1 — Exclusion of an outlier. Hemorrhagic transformation after 3 h MCAO in mice who received i.v. thrombolysis with 10 mg/kg human recombinant t-PA directly prior to reperfusion (middle, n = 5) and mice who were effectively anticoagulated to an INR of approximately 3 at the onset of cerebral ischemia (right, n = 5) compared to control mice with MCAO without pretreatment (left, n = 5). Hematoma volume detected by the hemoglobin assay is given in µl for each animal. The extreme value of 14.7 µl in the MCAO at INR 3 group was identified as a significant outlier by the Grubb's test and excluded from the statistical analysis. (TIF) [file pone.0026087.s001.tif]
